# Platelet count reduction during *in vitro* membrane oxygenation affects platelet activation, neutrophil extracellular trap formation and clot stability, but does not prevent clotting

**DOI:** 10.1177/0267659121989231

**Published:** 2021-01-21

**Authors:** Patrick Winnersbach, Jan Rossaint, Eva M. Buhl, Smriti Singh, Jonas Lölsberg, Matthias Wessling, Rolf Rossaint, Christian Bleilevens

**Affiliations:** 1 Department of Anaesthesiology, University Hospital RWTH Aachen University, Aachen, Germany; 2 Department of Anaesthesiology, Intensive Care and Pain Medicine, University Hospital Münster, Münster, Germany; 3 Institute of Pathology, Electron Microscopy Facility, RWTH Aachen University, Aachen, Germany; 4 DWI−Leibniz Institute for Interactive Materials Aachen, Aachen, Germany

**Keywords:** platelet activation, neutrophil extracellular trap (NET) formation, extracorporeal membrane oxygenation (ECMO), clot stability, *in vitro* test circuit

## Abstract

**Introduction:**

Due to improved technology and increased application the mortality during extracorporeal membrane oxygenation (ECMO) is constantly declining. Nevertheless, complications including haemorrhage or thrombus formation remain frequent. Local mitigation of coagulation within an ECMO system to prevent thrombus formation on ECMO components and optimizing systemic anticoagulation is an approach to reduce clotting and bleeding complications at once. Foreign surfaces of ECMO systems, activate platelets (PLTs), which besides their major role in coagulation, can trigger the formation of neutrophil extracellular traps (NETs) contributing to robust thrombus formation. The impact of a reduced PLT count on PLT activation and NET formation is of paramount importance and worth investigating.

**Methods:**

In this study platelet poor (PLT^–^) and native (PLT^+^) heparinized human blood was circulated in two identical *in vitro* test circuits for ECMO devices for 6 hours. PLT reduction was achieved by a centrifugation protocol prior to the experiments. To achieve native coagulation characteristics within the test circuits, the initial heparin dose was antagonized by continuous protamine administration.

**Results:**

The PLT^–^ group showed significantly lower platelet activation, basal NET formation and limited clot stability measured via thromboelastometry. Fluorescent and scanning electron microscope imaging showed differences in clot composition. Both groups showed equal clot formation within the circuit.

**Conclusions:**

This study demonstrated that the reduction of PLTs within an ECMO system is associated with limited PLT activation and NET formation, which reduces clot stability but is not sufficient to inhibit clot formation per se.

## Introduction

Extracorporeal membrane oxygenation (ECMO) often represents the final therapeutic option for ICU patients suffering from lung conditions, like acute respiratory distress syndrome (ARDS).^1^ A meta-analysis by Munshi et al.^2^ shows reduced mortality in severe ARDS patients receiving ECMO, compared to conservative therapy. Due to improvements in ECMO technology through the last decades, mortality during the therapy was reduced, from 90% in the 1970s.^3^ to 35% today.^4^ In particular, the increased use of ECMO in clinical routine during the H1N1 pandemic in 2009, led to decreased mortality rates ever since.^5^ However, the complication rate during ECMO still remains high, as major haemorrhage or cannula problems are frequent.^2^ In particular thrombus formation on the oxygenator membrane poses problems.^6^ During veno-arterial application of ECMO for cardiocirculatory support, the detachment of thrombi formed in an ECMO system, can lead to stroke by microembolism.^7^

A new experimental approach to reduce the risk of thrombus formation aims at bypassing platelets (PLTs) prior to the oxygenator via microfluidic cell sorting.^8^ The benefit of a PLT bypass, namely the reduction of PLT count within a membrane oxygenator under clinically relevant blood flow has not been shown yet but seems theoretically favourable. In addition to PLTs themselves, as key-players in thrombus formation, recent investigations show that platelet-neutrophil interactions and the release of neutrophil extracellular traps (NET) play a role in thrombus formation.^9,10^ Activated PLTs can trigger NET formation,^11,12^ which leads to thicker fibrin fibres and stronger clots.^13^ The influence of a PLT count reduction and its impact on PLT activation, NET and clot formation in components of ECMO circuits, especially on oxygenator membranes, has not been investigated in detail yet. Therefore, the aim of this study was to investigate the effect of a reduced platelet count in an *in vitro* test circuit for ECMO devices, previously described by Bleilevens et al.^14^ We reduced the PLT count of the blood samples, using a centrifugation protocol, and compared the impact on thrombus formation, coagulation activation and NET to blood with unaltered PLT count.

## Methods

### Blood donation and experimental groups

After informed consent and approval of the ethical committee of the University Hospital of the RWTH Aachen (file no EK355/16), we were allowed to withdraw 150 mL of blood (3 mL × 50mL syringe, primed with 3.75 IU/mL of heparin; LEO Pharma A/S, Ballerup, Denmark) from the median cubital vein of healthy volunteers. In this way blood donations from five healthy volunteers were used for five experiments. Each donation was split into two equal portions for simultaneous operation of the test circuits and direct comparison of two experimental groups (PLT^+^/PLT^–^).

Each portion was diluted, using crystalloid fluid (Sterofundin, B. Braun Melsungen AG, Melsungen, Germany), obtaining haemoglobin values between 7 and 8 g/dL, which matches values of critically ill ECMO-patients.

For the PLT^–^ group, a centrifugation protocol modified according to Bercovitz et al.^15^ was carried out. Whole blood (WB) was centrifuged at 400 *g* for 12 minutes, which resulted in the separation into platelet rich plasma (PRP) and red blood cells (RBCs). The PRP supernatant was transferred into a new vessel and the RBCs were put aside. PRP was again centrifuged at 4000 *g* for 10 minutes to obtain platelet poor plasma (PPP) and a platelet pellet, which was discarded. PPP was taken and merged with RBCs to form platelet poor whole blood (PLT^–^).

Blood for the PLT^+^ group remained unprocessed, and was stored in the dark, under slight motion. Directly after completion of the centrifugation process both test circuits were filled carefully, avoiding entrapped air and started simultaneously.

### Test circuit design

For each experiment two identical test circuits were assembled, consisting of three main parts: a centrifugal pump head (DeltaStream DP-II, Xenios AG, Heilbronn, Germany), an uncoated oxygenator (hilite 800 LT, Xenios AG, Heilbronn, Germany ) and a reservoir (extracorporeal membrane oxygenator R-14 Assist Reservoir, Medtronic Inc., Minneapolis, MN, USA). These parts are connected via PVC tubes (3/800, 1/400 and 1/1600, RAUMEDIC AG, Münchberg, Germany) to a closed loop, with a total filling volume of 150 mL.

Blood flow was measured via flow sensors (BioProTTTM Clamp-OnTM Transducer, em-tec GmbH, Finning, Germany), positioned between the pump head and the oxygenator.

During operation of the test circuits, a gas mix of 21% oxygen, 74% nitrogen and 5% carbon dioxide, with the total flow of 6 L/minute, was connected to each oxygenator, to achieve adequate blood gas values for an ECMO scenario. Carbon dioxide was added to simulate its release by living cells, which are missing in this *in vitro* model, as 4%–5% represent the physiological CO_2_ concentration in human lungs. A steady temperature of 37 °C within the circuit, was assured via a heat exchanger and continuous temperature monitoring. The test circuits contain two connections to syringe pumps, for continuous injection of nutrient-solution and protamine (MEDA Pharma GmbH & Co. KG, Bad Homburg, Germany).

### Circulation and haemodynamics

To achieve clinically relevant haemodynamic properties in the test circuits, the blood flow was constantly adjusted to 150 mL/minute, which is equivalent to the circulation of one total loop volume per minute. That reflects the cardiac output of 4–5 L/minute in an ECMO scenario, which equals the average total blood volume of an adult.

An adjustable clamp, which exerted pressure on the reservoir, was used to mimic an appropriate pressure of ⩾ 75 mmHg within both test circuits.

Volume loss, due to blood sampling and evaporation of 0.046 mL/minute/L gas flow, as described previously,^16^ was covered by a continuous application of nutrient solution. The nutrient solution consists of phosphate, adenine, glucose, guanosine saline and mannitol and was described previously as a preservation solution of red blood cells for *in vitro* test circuits.^16^

### Anticoagulation protocol

Initially, after donation the blood was anticoagulated with 3.75 IU/mL of heparin, resulting in a total amount of 281.25 IU heparin per test circuit. As the native heparanase function is missing *in vitro*, continuous protamine supply was started simultaneously with circulation and maintained for 2 hours. As, in theory, one IU of protamine antagonizes one IU of heparin, 140.63 IU protamine were added in total. More precisely, protamine (14.06 IU/ml) was administered with a rate of 5 ml/hour for 2 hours.

Thus, 50% of the initial heparin effect was antagonized and coagulation activation by the foreign surface of the ECMO parts and clotting was enabled.

In contrast to common clinical practice, uncoated oxygenator membranes were used to accelerate clot formation.

Altogether the chosen protocol, comprising the administration of protamine and the use of uncoated oxygenator membranes, leads to an accelerated scenario, which complies with the limited time frame of *in vitro* test circuit trials.

### Blood sampling and analysis

**Figure 1.**
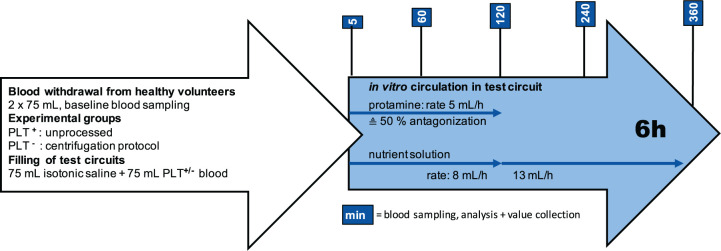
Flow chart. White arrow: Overview of blood preparation and experimental groups prior to circulation. Blue arrow: Points in time for blood sampling and infusions during *in vitro* circulation in the test circuit.

Blood sampling was performed at different time points, as shown in Figure 1. Blood was collected into citrate respectively serum gel collection tubes (S-Monovette, Sarstedt Inc., Nümbrecht, Germany). Blood gas analysis was performed on an ABL90-flex analyser (Radiometer Inc., Fichtenhain, Germany), a haemogram was measured on an automated cell counter (Sysmex XT-2000iVet, SysmexCorporation, Kobe, Japan). The concentration of free haemoglobin (fHGB) was determined spectroscopically on a microplate reader (ELx800; BioTek Inc, Bad Friedrichshall, Germany) at 540 and 680 nm wavelength, using the potassium-cyanide method with a commercially available kit (Ref004001-0250, Biorapid Inc., Freiburg, Germany).

### Fluorescence-activated cell sorting

To determine the ratio of activated PLTs, 20 µl citrated blood was used to detect cell surface expression of P-selectin (CD62P+), as described previously.^17^ Briefly, 1:50 diluted whole blood samples from the loop were incubated with fluorescein isothiocyanate (FITC)-conjugated and phycoerythrin (PE)-conjugated CD61 and CD62P (MCA2263F, MCA2418PE, BioRad Inc., Feldkirchen, Germany) or their appropriate isotype controls (MCA928F, MCA 928PE, BioRad Inc., Feldkirchen, Germany) for 15 minutes at room temperature as described previously. After staining, PLTs were fixed for analysis (Cell Fix; BD Biosciences, Heidelberg, Germany), using a Flow Cytometer (BD FACSCanto, BD Bioscineces, San Jose, CA, USA).

### Thromboelastometry

Thromboelastometry was performed on a ROTEM analyser (Werfen GmbH, München, Germany) to determine the Maximum Clot Firmness (MCF), in the extrinsic coagulation pathway, using EXTEM-reagents and 900 µl citrated blood.

### Quantification of neutrophil extracellular traps formation

A myeloperoxidase-DNA enzyme-linked immunosorbent assay was performed to measure the quantity of NET structures in serum, as described and conducted previously.^11,18^

### Fluorescence microscopy

Clots from both experimental groups were extracted from the reservoir and the pump heads of the test circuits and subsequently fixed in 2% formalin. Clots were embedded in an optimal cutting temperature compound (O.C.T., Tissue Tek Inc., Munich, Germany), frozen to -80°C and cut in 5 µm thick sections using a cryotome. The tissue sections were mounted on glass slides, fixed with ice-cold methanol, washed with phosphate buffered saline and blocked with 3% BSA/T-BST. Primary staining was performed with PE-coupled CD41 antibody (clone MWReg30, dilution 1:500, Biolegend), FITC-coupled erythrocyte antibody (clone Ter119, dilution 1:500, Biolegend) and primary anti-Histone H2A antibody (sc-8648, 1:500, Santa Cruz). Secondary staining was performed with anti-goat Alexa568-coupled secondary antibody (dilution 1:1000, Invitrogen). After mounting of cover slips images were obtained using a LionHeart XF Imager (BioTek Instruments).

### Scanning electron microscopy

After termination of circulation in the test circuit, blood was drained from the oxygenator, rinsed with saline solution and fixed in 2% formalin. After fixation the oxygenators were carefully cut open and few polymethylpentene fibres, from each quadrant, were extracted for scanning electron microscopy (SEM). Samples were dehydrated in an ascending ethanol series (30%, 50%, 70%, 90% and 100%). The final drying step was performed in a critical point dryer (E3100, Quorum Technologies, Lewes, UK). Dried samples were sputter-coated (EM SCD500, Leica, Wetzlar, Germany) with a 10 nm gold palladium layer and analysed using an environmental scanning electron microscope (ESEM XL 30 FEG, FEI, Eindhoven, The Netherlands) in a high vacuum environment using an acceleration voltage of 10 kV.

### Statistical analysis

Presented data are shown as the mean ± SD. Using GraphPad Prism software (GraphPad Prism version 9.0.0 for MacOS, GraphPad Software, San Diego, California USA) a two-way ANOVA with Sidak correction for multiple comparisons and a confidence interval of 95% was performed to verify differences at single time points between the two paired groups (PLT^+^/PLT^−^). If the calculated p-value was < 0.05, the results were regarded as significantly different. GraphPad Prism was also used to design the graphs.

## Results

### Cell count

**Figure 2.**
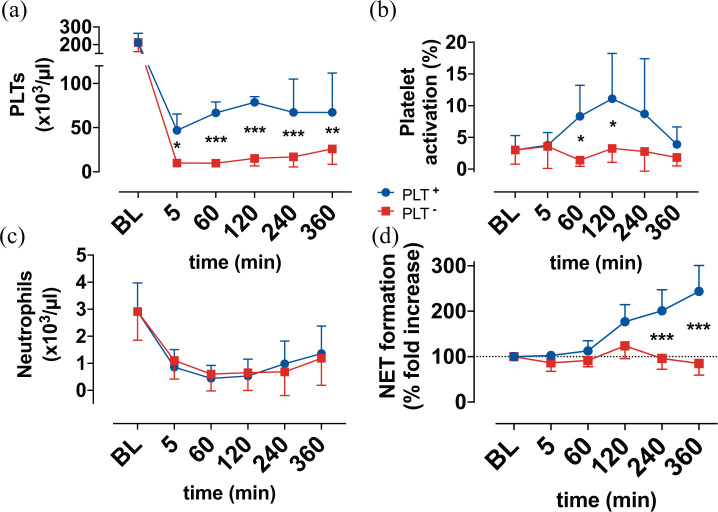
Display of: (a) platelets (PLTs), (b) platelet activation (CD62+/CD61+), (c) neutrophils and (d) neutrophil extracellular trap formation (NET formation) over time. Baseline (BL). *p < 0.05. **p < 0.01.***p < 0.001.

The baseline PLT count (Figure 2(a)) was 212 ± 52 × 10^3^/μl. After dilution with crystalline solution, and 1 hour of circulation in the test circuit, the PLT count in the PLT^+^ group decreased to 67 ± 12 × 10^3^/μl, compared to 10 ± 4 × 10^3^/μl in the PLT^–^ group. During circulation the PLT count in both groups raised gradually, with a maximum count of 79 ± 6 × 10^3^/μl in the PLT^+^ group and 26.0 ± 18 × 10^3^/μl in the PLT^–^ group, respectively.

**Figure 3.**
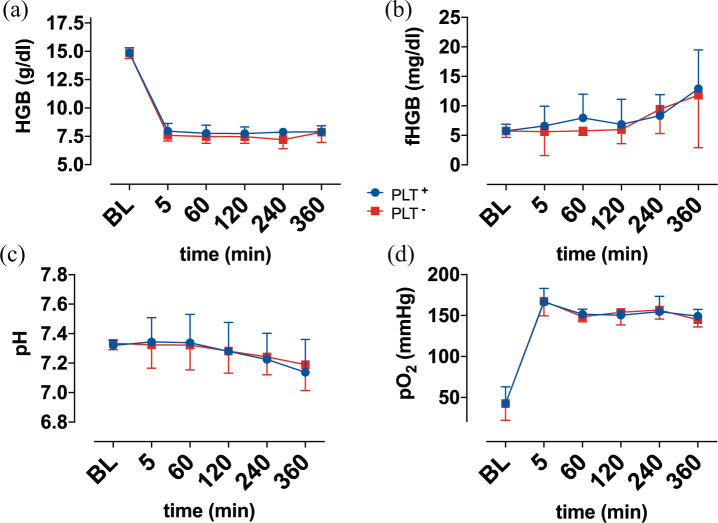
Display of: (a) haemoglobin (HGB), (b) free haemoglobin (fHGB), (c) pH and (d) pO_2_ over time. Baseline (BL).

Mean haemoglobin concentration of the blood donation was 14.8 ± 0.5 g/dl, which was diluted to values between 7 and 8 g/dl in both groups and remained at this level, as shown in Figure 3(a).

The concentration of fHGB increased in both groups from 5.8 ± 1.1 mg/dl, without significant differences between the groups (Figure 3(b)).

The neutrophils count dropped after dilution in both groups from 2.9 ± 1.1 × 10^3^/μl to 0.9 ± 0.7 × 10^3^/μl (PLT^+^) and 1.1 ± 0.7 × 10^3^/μl (PLT^−^), showed a slight increase until the end of the experiments without differences between the groups (Figure 2(c)).

### Fluorescence-activated cell sorting

PLT activation (Figure 2(b)) in the PLT^–^ group remained at baseline levels for the whole experiments, whereas the activation was significantly increased in the PLT^+^ group after 60 minutes of circulation, before it dropped back to comparable level with the PLT^–^ group at the end of the experiments (3.9.4 ± 2.8% (PLT^+^) vs 1.8 ± 1.3% (PLT^−^)).

### NET formation

NET formation (Figure 2(d)) increased over time to a maximum of 244 ± 57% of the baseline level at the end of the experiments in the PLT^+^ group, whereas the NET formation in the PLT^–^ group remained on baseline levels.

### Blood gas analysis

Initial venous pO_2_ (Figure 3(d)) at baseline was 42.6 ± 20.4 mmHg. Due to oxygenation in the test circuit, pO_2_ values increased over 150 mmHg in both groups and remained at this level.

Elimination of CO_2_ (data not shown) by the oxygenator, in either group, lead to a pCO_2_ of 34.1 ± 0.2 mmHg after 5 minutes, from an initial value of 49.1 ± 9.2 mmHg. Until the end of circulation, values slightly decreased to 30.6 ± 0.2 mmHg.

Within the first hour of circulation pH remained stable at baseline levels 7.33 ± 0.01 and decreased to 7.16 ± 0.04 at the end of experiments, in both groups, as shown in Figure 3(c).

### Operating parameters

The blood flow within the circuit was held constant at 150 mL/minute in both groups. The pump’s power, measured in revolutions per minute (rpm) of the pump’s rotor, increased from 1246 ± 62 to 1280 ± 90 rpm. There were no significant differences between both groups. The loss of pressure due to the oxygenator’s resistance increased, in both groups, in accord with the power of the pump (data not shown).

### Clot formation

**Figure 4.**
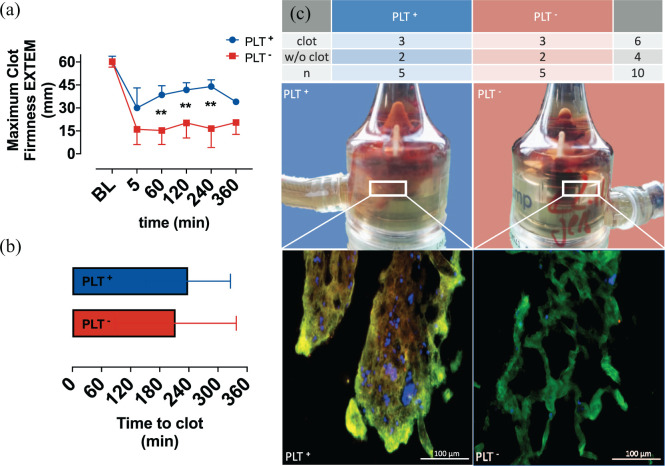
Display of: (a) Mean clot firmness of extrinsic coagulation pathway (MCF EXTEM) and (b) time until first signs (an increase of pump’s power to maintain flow) of coagulation within the test circuit (Time to clot). (c) Table summarizing experiments with (clot) and without (c/o clot) clotting within the test circuits in the experimental groups: platelet poor whole blood (PLT^–^) and whole blood (PLT^+^). Images of pump heads including clots. Representative fluorescence microscopy images of histological clot sections excised from the pump heads (blue: platelets anti-CD41; green: erythrocytes anti-TER119; red: histones in NETs anti-H2A). Baseline (BL). **p < 0.01.

In both groups fulminant clot formation was detected in three out of five experiments. The time of circulation, until the first signs of clotting occurred (Time to clot), also did not show significant differences between both groups (Figure 4(b)). Pictures of the pump heads, including immunofluorescence images of clot morphology within the pump heads are shown in Figure 4(c).

### Thromboelastometry

Thromboelastometry indicated significant differences in clot formation and stability, with a peak value of the MCF after 240 minutes in the PLT^–^ group 16.5 ± 12.4 mm, compared to 44.0 ± 4.4 mm in the PLT^+^ group, as shown in Figure 4(a).

### Scanning Electron Microscopy

**Figure 5.**
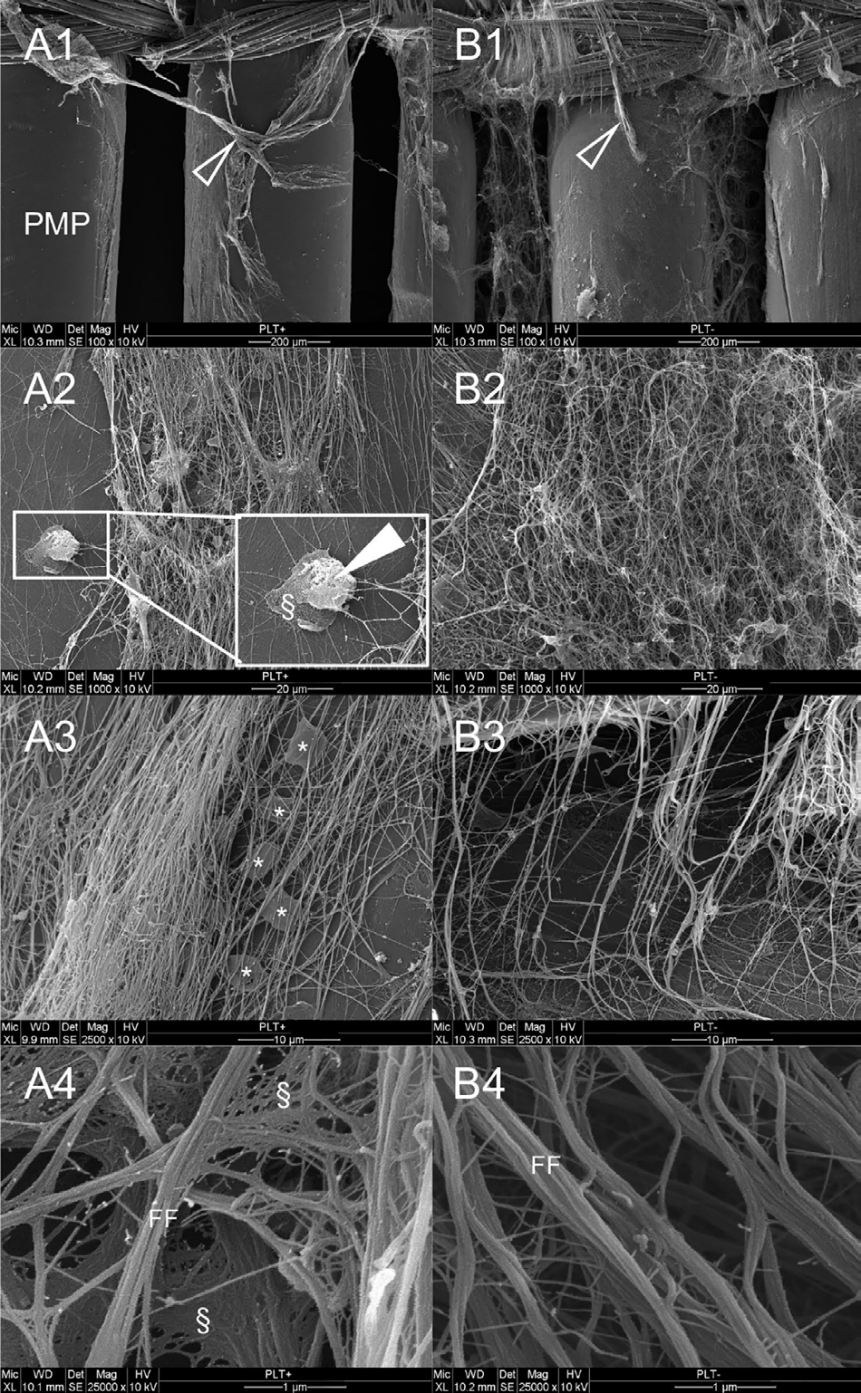
Representative scanning electron microscopy images of polymethylpentene fibres of oxygenators after 360 minutes *in vitro* blood circulation: (A) PLT^+^ group, (B) PLT^–^ group. **1** 100-fold magnification; **2** 1000-fold magnification; **3** 2500-fold magnification; **4** 25,000-fold magnification. PMP: Polymethylpentene fibres of the oxygenator membrane; Arrow: Fibrin deposit; Filled arrow: Neutrophil granulocyte; § Neutrophil extracellular traps; * Activated, flat platelets; FF: Fibrin fibres.

SEM images (Figure 5) show the ultrastructural architecture of the fibrin clots on polymethylpentene fibres. In the PLT^+^ group (A) the fibrin fibres are straight, dense and predominantly parallel structured, as seen at 1000- and 2500-fold magnification (A2–A4). Within the fibrin fibres are NETs, recognizable as laminar structures spanning between the fibres. A close-up to the fibres reveals a rough appearing fibre surface indicating adherent protein aggregates (A4).

In comparison to that, the fibre deposits in the PLT^–^ group (B) are less dense structured (B2–B4) and the fibre surface is smooth (B4). Furthermore, less NET formation is observed.

## Discussion

In this study we circulated blood from healthy human volunteers with diverging PLT concentrations in two similar constructed test circuits for 6 hours and compared their thrombotic properties. We investigated the influence of a reduced PLT count on coagulation and thrombus formation, in an *in vitro* ECMO test circuit. In contrast to our hypothesis the experiments could not show reduced clot formation in the PLT^–^ group. In both groups three out of five experiments showed clot formation within the circuit, and also the time span until the onset of coagulation activation showed no significant differences.

However, analysis of clot stability, PLT activation and NET formation revealed major variations between both experimental groups. Thromboelastometry showed significantly reduced clot firmness, as detected by MCF measurements after 60, 120 and 240 minutes in the PLT^–^ group, which indicates a decreased clot stability. Significantly higher levels of PLT activation could be seen in the PLT^+^ group, after 60 minutes of circulation, followed by substantial increase in NET formation in contrast to persistent values at baseline levels in the PLT^–^ group.

The clots extracted from both loops after termination showed macroscopic differences as well as differences in their composition and structure, visualized by fluorescent microscopic and SEM imaging.

The initial PLT drop in the PLT^+^ group can be explained by the dilution with crystalloid solution and the adhesion of PLTs on the oxygenator membrane. This phenomenon, including the detachment of PLTs from the oxygenator membrane during circulation leads to a renewed increase of the PLT count after 60 minutes, was previously described by Hennessy et al.^19^ Furthermore, the experimental setting proved to ensure mostly stable conditions concerning blood gas values, operational settings and temperature.

Haemolysis indicated by the increase of fHGB after 240 minutes of circulation, is within the same range as previously performed *in vitro* tests using these test circuits,^14,16^ and is not uncommon in clinical ECMO administration.^20^

PLT activation after 5 minutes of circulation is similar in both groups, which demonstrates that the prior centrifugation has no measurable impact on the activation of the remaining PLTs in the PLT^–^ group. Higher levels of PLT activation (expression of CD62P), starting after 60 minutes of circulation, in the PLT^+^ group can be explained by a potentiation of PLT stimulation, due to a larger number (higher concentration) of PLTs in immediate surroundings. PLT activation leads to further PLT activation via secretion of signalling molecules. Secreted adenosine diphosphate and serotonin activates resting PLTs by binding to receptors on their surface. Thus activated PLTs lead to an amplification of PLT activation.^21^

Prior studies demonstrated that activated PLTs trigger NET formation by means of direct platelet-neutrophil interactions and soluble mediators.^12^ This study reinforces these findings, as we observe that increased PLT activation in the PLT^+^ group is followed by higher levels of NET formation at subsequent time points. The delay in the increase of NET consecutively to PLT activation, can be explained by the duration of the NET formation process. Decondensation of chromatin and release of NET by neutrophils is an active and time-consuming process.^22^ In contrast the PLT^–^ group showed less PLT activation and nearly no NET formation.

Previous *in vivo* and *in vitro* studies showed that the PLT count is positively correlated with MCF in thromboelastometry analysis, which matches with our results.^23^ In addition to a reduced PLT count, less NET formation in the PLT^–^ group could contribute to an impaired clot stability which was indicated by lower MCF. In contrast the PLT^+^ group even shows a slightly increasing MCF, after 60–240 minutes, concordantly with a sharp rise of NETs. This observation can be explained as extracellular DNA and decondensed chromatin, components of NET, support the fibrin structure resulting in higher clot stability and rigidity.^13^

The notion of reduced clot firmness in the PLT^–^ group is also supported by the results of immunofluorescence imaging and electron microscopy, where blood clots, stained with fluorescent labelled antibodies against PLTs and NET structures showed sparsely scattered PLTs and minimal NET content of the blood clots. SEM images showed loose and undirected fibrin fibres in the PLT^–^ group in contrast to the PLT^+^ group, which shows straight, dense fibres with NETs in between.

As an outlook, we hypothesize that inhibiting the axis of PLT activation and subsequent NET formation within an ECMO system, might support the effort in a clinical ECMO scenario, to reduce the essential systemic anticoagulation of ECMO patients, to minimize the bleeding risk, without enhancing the risk for oxygenator thrombosis.

Recent studies indicate that the local application of nitric oxide (NO) is feasible and effective. ^24^ NO is a potent, short-acting inhibitor of PLT activation,^25^ that allows local prevention of adhesion and aggregation within an ECMO circuit.^24,26^ Inhaled NO is commonly used in ARDS for the reduction of pulmonary-artery pressure.^27^

DNAse disassembles NET^28^ and can prevent vascular occlusion due to NET formation.^29,30^ DNAse was previously used in clinical trials without significant adverse events,^31,32^ furthermore inhaled DNAse is used as treatment strategy in cystic fibrosis.^33^ The possibilities for an application of NO and DNAse and their individual or combined efficacy, as local anticoagulation strategy within an ECMO system, requires further investigation, but seems reasonable.

### Limitations

By means of the used centrifugation protocol it was not possible to completely remove PLTs in the PLT^–^ group. Still it was possible to generate significant differences in PLT count between the examined groups. The effect of the residual PLTs in the PLT^–^ group and its extent on coagulation remains unclear.

The number of experiments (n=5) was small, nevertheless we were able to show significant differences concerning relevant parameters, as NET formation or MCF.

The study remains an *in vitro* study with limited validity for *in vivo* translation or application.

As cells and organs are absent in this *in vitro* setting important blood components like coagulation factors, cytokines and electrolytes are not reproduced, which results in an altered homeostasis. However, parameters like temperature, blood gases or pressure within the system, were kept within a range not uncommon for critical ill patients.

## Conclusion

In conclusion this study demonstrates that the reduction of PLTs within an ECMO system is associated with limited PLT activation and subsequent NET formation, which leads to lower clot stability, but is not sufficient to inhibit clot formation per se.

## References

[1] Brodie D Bacchetta M. Extracorporeal membrane oxygenation for ARDS in adults. N Engl J Med 2011; 365: 1905–1914. 22087681 10.1056/NEJMct1103720

[2] Munshi L Walkey A Goligher E , et al. Venovenous extracorporeal membrane oxygenation for acute respiratory distress syndrome: a systematic review and meta-analysis. Lancet Respir Med 2019; 7: 163–172. 30642776 10.1016/S2213-2600(18)30452-1

[3] Zapol WM Snider MT Hill JD , et al. Extracorporeal membrane oxygenation in severe acute respiratory failure. A randomized prospective study. JAMA 1979; 242: 2193–2196. 490805 10.1001/jama.242.20.2193

[4] Combes A Hajage D Capellier G , et al. Extracorporeal membrane oxygenation for severe acute respiratory distress syndrome. N Engl J Med 2018; 378: 1965–1975. 29791822 10.1056/NEJMoa1800385

[5] Zangrillo A Biondi-Zoccai G Landoni G , et al. Extracorporeal membrane oxygenation (ECMO) in patients with H1N1 influenza infection: a systematic review and meta-analysis including 8 studies and 266 patients receiving ECMO. Crit Care 2013; 17: R30. 10.1186/cc12512 PMC4057025 23406535

[6] Lubnow M Philipp A Foltan M , et al. Technical complications during veno-venous extracorporeal membrane oxygenation and their relevance predicting a system-exchange – retrospective analysis of 265 cases. PLoS One 2014; 9: e112316. 10.1371/journal.pone.0112316 PMC4251903 25464516

[7] Lo Coco V Lorusso R Raffa GM , et al. Clinical complications during veno-arterial extracorporeal membrane oxigenation in post-cardiotomy and non post-cardiotomy shock: still the achille’s heel. J Thorac Dis 2018; 10: 6993–7004. 30746245 10.21037/jtd.2018.11.103 PMC6344687

[8] Bleilevens C Lölsberg J Cinar A , et al. Microfluidic cell sorting: towards improved biocompatibility of extracorporeal lung assist devices. Sci Rep 2018; 8: 8031. 10.1038/s41598-018-25977-6 PMC5966447 29795137

[9] von Brühl M-L Eckart A Coletti R , et al. Monocytes, neutrophils, and platelets cooperate to initiate and propagate venous thrombosis in mice in vivo. J Exp Med 2012; 209: 819–835. 22451716 10.1084/jem.20112322 PMC3328366

[10] Gould TJ Vu TT Swystun LL , et al. Neutrophil extracellular traps promote thrombin generation through platelet-dependent and platelet-independent mechanisms. Arterioscler Thromb Vasc Biol 2014; 34: 1977–1984. 25012129 10.1161/ATVBAHA.114.304114

[11] Rossaint J Herter JM Van Aken H , et al. Synchronized integrin engagement and chemokine activation is crucial in neutrophil extracellular trap-mediated sterile inflammation. Blood 2014; 123: 2573–2584. 24335230 10.1182/blood-2013-07-516484

[12] Rossaint J Zarbock A. Platelets in leucocyte recruitment and function. Cardiovasc Res 2015; 107: 386–395. 25712962 10.1093/cvr/cvv048

[13] Longstaff C Varjú I Sótonyi P , et al. Mechanical stability and fibrinolytic resistance of clots containing fibrin, DNA, and histones. J Biol Chem 2013; 288: 6946–6956. 23293023 10.1074/jbc.M112.404301 PMC3591605

[14] Bleilevens C Hill A Grzanna T , et al. In vitro head-to-head comparison of anticoagulation properties of two heparin brands in a human blood miniature mock loop. Interact Cardiovasc Thorac Surg 2019; 28: 120–127. 30010987 10.1093/icvts/ivy206

[15] Bercovitz RS Brenner MK Newman DK. A whole blood model of thrombocytopenia that controls platelet count and hematocrit. Ann Hematol 2016; 95: 1887–1894. 27515424 10.1007/s00277-016-2777-9 PMC6055520

[16] Bleilevens C Grottke O Tillmann S , et al. Twelve hours in vitro biocompatibility testing of membrane oxygenators. ASAIO J 2015; 61: 548–555. 26273935 10.1097/MAT.0000000000000252

[17] Bleilevens C Grottke O Groening S , et al. Septic porcine blood does not further activate coagulation during in vitro membrane oxygenation. Eur J Cardiothorac Surg 2017; 51: 449–456. 27806995 10.1093/ejcts/ezw345

[18] Caudrillier A Kessenbrock K Gilliss BM , et al. Platelets induce neutrophil extracellular traps in transfusion-related acute lung injury. J Clin Invest 2012; 122: 2661–2671. 22684106 10.1172/JCI61303 PMC3386815

[19] Hennessy VL Jr Hicks RE Niewiarowski S , et al. Function of human platelets during extracorporeal circulation. Am J Physiol 1977; 232: H622–H628. 10.1152/ajpheart.1977.232.6.H622 18017

[20] O’Brien C Monteagudo J Schad C , et al. Centrifugal pumps and hemolysis in pediatric extracorporeal membrane oxygenation (ECMO) patients: an analysis of Extracorporeal Life Support Organization (ELSO) registry data. J Pediatr Surg 2017; 52: 975–978. 28359588 10.1016/j.jpedsurg.2017.03.022

[21] Jurk K Kehrel BE. Platelets: physiology and biochemistry. Semin Thromb Hemost 2005; 31: 381–392. 16149014 10.1055/s-2005-916671

[22] Zucoloto AZ Jenne CN. Platelet-neutrophil interplay: insights into Neutrophil Extracellular Trap (NET)-driven coagulation in infection. Front Cardiovasc Med 2019; 6: 1–8. 31281822 10.3389/fcvm.2019.00085 PMC6595231

[23] Shenkman B Einav Y Livnat T , et al. In vitro evaluation of clot quality and stability in a model of severe thrombocytopenia: effect of fibrinogen, factor XIII and thrombinactivatable fibrinolysis inhibitor. Blood Transfus 2014; 12: 78–84. 24333083 10.2450/2013.0068-13 PMC3926734

[24] Roberts TR Neufeld MJ Meledeo MA , et al. A metal organic framework reduces thrombus formation and platelet aggregation ex vivo. J Trauma Acute Care Surg 2018; 85: 572–579. 29787534 10.1097/TA.0000000000001982

[25] Radomski MW Moncada S. The biological and pharmacological role of nitric oxide in platelet function. Adv Exp Med Biol 1993; 344: 251–264. 7516113 10.1007/978-1-4615-2994-1_20

[26] Skrzypchak AM Lafayette NG Bartlett RH , et al. Effect of varying nitric oxide release to prevent platelet consumption and preserve platelet function in an in vivo model of extracorporeal circulation. Perfusion 2007; 22: 193–200. 18018399 10.1177/0267659107080877

[27] Rossaint R Falke KJ Lopez F , et al. Inhaled nitric oxide for the adult respiratory distress syndrome. N Engl J Med 1993; 328: 399–405. 8357359 10.1056/NEJM199302113280605

[28] Hakkim A Fürnrohr BG Amann K , et al. Impairment of neutrophil extracellular trap degradation is associated with lupus nephritis. Proc Natl Acad Sci U S A 2010; 107: 9813–9818. 20439745 10.1073/pnas.0909927107 PMC2906830

[29] Jiménez-Alcázar M Rangaswamy C Panda R , et al. Host DNases prevent vascular occlusion by neutrophil extracellular traps. Science 2017; 358: 1202–1206. 29191910 10.1126/science.aam8897

[30] Brill A Fuchs TA Savchenko AS , et al. Neutrophil extracellular traps promote deep vein thrombosis in mice. J Thromb Haemost 2012; 71: 3831–3840. 10.1111/j.1538-7836.2011.04544.x PMC3319651 22044575

[31] Jorch SK Kubes P. An emerging role for neutrophil extracellular traps in noninfectious disease. Nat Med 2017; 23: 279–287. 28267716 10.1038/nm.4294

[32] Davis JC Manzi S Yarboro C , et al. Recombinant human Dnase I (rhDNase) in patients with lupus nephritis. Lupus 1999; 8: 68–76. 10025601 10.1191/096120399678847380

[33] Gray RD McCullagh BN McCray PB. NETs and CF lung disease: current status and future prospects. Antibiotics 2015; 4: 62–75. 27025615 10.3390/antibiotics4010062 PMC4790323

